# International Medical Congress

**Published:** 1876-03

**Authors:** S. D. Gross

**Affiliations:** President; Philadelphia


### Editorial.

### INTERNATIONAL MEDICAL CONGRESS, PHILADELPHIA, 1876.

SEPTEMBER 4TH 9TH.

We make the following extracts from the Preliminary Programme issued by the Commission :

The International Medical Congress will be formally opened at noon, on Monday, the 4th day of September, 1876, in the University of Pennsylvania.

The following addresses will be delivered before the Congress in general meeting.

Address on Medicine, by Austin Flint, M. D., Professor of Practice of Medicine in Bellevue Hospital Medical College, New York.

Address on Hygiene and Preventive Medicine, by Henry I. Bowditch, M. D., President of State Board of Health of Massachusetts.

Address on Surgery, by Paul F. Eve, M. D., Professor of Operative and Clinical Surgery in the University of Nashville.

Address on Obstetrics, by Theophilus Parvin, M. D., Professor of Obstetrics in the College of Physicians and Surgeons of Indiana.

Address on Medical Chemistry and Toxicology, by Theodore G. Wormley, M. D., Professor of Chemistry in Starling Medical College, Columbus, Ohio.

Address on Medical Biography, by J, M. Toner, M. D., of Washington, D. C.

Address, by Dr. Hermann Lebert, Professor of Clinical Medicine in the University of Breslau.

Address on Medical Education and Medical Institutions, by Nathan S. Davis, M. D., Professor of Principles and Practice of Medicine in Chicago Medical College.

Address on Medical Literature, by Lunsford P. Yandell, M. D., late Professor of Physiology in the University of Louisville.

Address on Mental Hygiene, by John P. Gray, M. D., Superintendent and Physician to the New York State Lunitic Asylum, Utica, New York.

Address on Medical Jurisprudence, by Stanford E. Chaille, M. D., Professor of Physiology and Pathological Anatomy in the University of Louisiana.

The Congress will be divided into the following sections in which discussions upon various scientific subjects will be held :

I. Medicine. II. Biology. III. Surgery. IV. Dermatology and Syphiology. V. Obstetrics. VI. Ophthalmology. VII. Otology, VII Sanitary Science. IX. Mental Diseases.

The gentlemen who have been named to open the discussions in these various sections are all well known and will present papers which will be worthy of the consideration of the Congress. As the programme published is but a preliminary one, the Commission request gentlemen intending to make communications upon scientific subjects or to participate in any of the debates, to notify them before the first of August that place may be assigned them on the programme.

In order to facilitate debate there will be published on or about June 1st the outlines of the opening remarks by the several reporters. Copies may be obtained on application to the Corresponding Secretaries.

The volume of Transactions will be published as soon as practicable after the adjournment of the Congress.

The Public Dinner of the Congress will be given on Thursday, September 7th, at 6.30 P. M.

The registration book will be open daily from Thursday, Aug. 31, from 12 to 3, P. M., in the Hall of the College of Physicians, N. E. corner 13th and Locust Streets. Credentails must in every case be presented.

The registration fee (which will not be required from foreign members) has been fixed at Ten Dollars, and will entitle the member to a copy of the Transactions of the Congress.

Gentlemen attending the Congress can have their correspondence directed to the care of the College of Physicians of Philadelphia, N. E. corner of Locust and Thirteenth Sts., Philadelphia, Pennsylvania.

There is every reason to believe that there will be ample hotel accommodation' at reasonable rates, for all strangers visiting Philadelphia in 1876. Further information may be obtained by addressing the Corresponding Secretaries.

All communications must be addressed to the appropriate Secretaries at Philadelphia.

S. D. GROSS, M. D.,
President.

WILLIAM B. ATKINSON, M. D., 1400 Pine Street, Recording Secretary.
WILLIAM GOODELL, M. D., 20th and Hamilton Sts.,
DANIEL G. BRINTON, M. D., 115 S. 7th Street,
American Corresponding Secretaries.
RICHARD J. DUNGLISON, M. D., 814 N. 16th Street.
R. M. BERTOLET, M. D., 113 S. Broad Street.
Foreign Corresponding Secretaries.

Philadelphia, March, 1876.

